# Prevalence of Malocclusion among 10-12-year-old Schoolchildren in Kozhikode District, Kerala: An Epidemiological Study

**DOI:** 10.5005/jp-journals-10005-1333

**Published:** 2016-04-22

**Authors:** Retna Kumari Narayanan, MT Jeseem, TV Anupam Kumar

**Affiliations:** 1 Professor and Head, Department of Pedodontics and Preventive Dentistry Government Dental College, Kozhikode, Kerala, India; 2 Postgraduate Student, Department of Pedodontics and Preventive Dentistry Government Dental College, Kozhikode, Kerala, India; 3 Associate Professor, Department of Pedodontics and Preventive Dentistry Government Dental College, Kozhikode, Kerala, India

**Keywords:** Crossbite, Malocclusion, Prevalence.

## Abstract

**Background:** A malocclusion is an irregularity of the teeth or a malrelationship of the dental arches beyond the range of what is accepted as normal.

**Objectives:** To determine the prevalence of malocclusion in children aged 10-12 years in Kozhikode district of Kerala, South India.

**Materials and methods:** A descriptive cross-sectional study was conducted among schoolchildren aged 10-12 years in six schools in Kozhikode district of Kerala, South India. A total of 2,366 children satisfied the inclusion criteria. Occlusal characteristics like crossbite, open bite, deep bite, protrusion of teeth, midline deviations, midline diastema and tooth rotation were recorded. The data were tabulated and analyzed using Chi-square test.

**Results:** The results revealed that the overall prevalence of malocclusion was 83.3%. Of this, 69.8% of the children had Angle’s class I malocclusion, 9.3% had class II malocclusion (division 1 = 8.85%, division 2 = 0.5%) and 4.1% had class III malocclusion; 23.2% showed an increased overjet (>3 mm), 0.4% reverse overjet, 35.6% increased overbite (>3 mm), 0.29% open bite, 7.2% crossbite with 4.6% crossbite of complete anterior teeth, 63.3% deviation of midline, 0.76% midline diastema and 3.25% rotated tooth. No significant differences in gender distributions of malocclusions were noted except for increased overjet and overbite.

**Conclusion:** There is high prevalence of malocclusion among schoolchildren in Kozhikode district of Kerala. Early interception and early correction of these malocclusions will eliminate the potential irregularities and malpositions in the developing dentofacial complex.

**How to cite this article:** Narayanan RK, Jeseem MT, Kumar TVA. Prevalence of Malocclusion among 10-12-year-old Schoolchildren in Kozhikode District, Kerala: An Epidemiological Study. Int J Clin Pediatr Dent 2016;9(1):50-55.

## INTRODUCTION

Malocclusion is a continuum ranging from an ideal occlusion to considerable deviation from normal.^1^ It has large impact on individual and society in terms of discomfort, quality of life and social and functional limitations. The etiology of malocclusion may be genetic, environmental or more commonly a combination of them. In addition, local factors such as adverse oral habits, anomalies in number, form and developmental position of teeth can also cause malocclusion.^2^ Early interception and early correction of these malocclusions will prevent their progression to its full form and will exclude factors interfering with the regular development of the dental arches.^3^

In India, the prevalence of malocclusion varies from 20-43%.^4^ Presently, there is insufficient literature regarding the prevalence of malocclusion in Kerala state of India. Therefore, the present study was carried out to determine the prevalence of malocclusion and associated variables among schoolchildren in Kozhikode district of Kerala.

## AIMS AND OBJECTIVES

- To determine the prevalence of malocclusion among 10 to 12-year-old schoolchildren.
- To identify the proportion of various types of malocclusion.
- To determine the proportion of different variables of malocclusions like crossbite, open bite, protrusion of teeth, deep bite, midline diastema, midline deviations and rotation of teeth.

## MATERIALS AND METHODS

A descriptive cross-sectional study was conducted to assess the prevalence of malocclusion among schoolchildren aged 10-12 years in six schools in Kozhikode district of Kerala. The study was undertaken by the Department of Pedodontics and Preventive Dentistry, Government Dental College, Kozhikode and was conducted for a period of 1 year.

The permission for conducting the study was taken from the District Education Officer, Kozhikode. The necessary information, such as names of all schools in Kozhikode district, their addresses and total number of students studying in each division in each school was obtained from the Education Council for the construction of a sample frame. Considering an average of 400 students from each school, six schools were randomly selected using cluster sampling method. To make up the estimated sample size, a total of 2,424 students were examined, among which 2,366 children satisfied the inclusion criteria.

Ethical clearance was obtained from the Ethical Committee of Government Dental College. Permission from the school authorities and consent from parents of children examined were obtained before the commencement of the study.

### Inclusion Criteria

- The children of the age group of 10-12 years who were present on the day of examination with the informed consent of their parents
- Children who had all the permanent first molars erupted.

### Exclusion Criteria

- Previous history of orthodontic treatment
- Craniofacial anomalies
- Uncooperative child
- Medically compromised child.

### Examination of the Children

The oral examination was conducted by a single trained examiner using disposable gloves, standard mouth mirror and probe. The children were examined while seated on chair with good natural light/artificial illumination during class hours in a predetermined order. Each child was examined using the World Health Organization (WHO) criteria for oral health assessment. The assessment of the dental occlusion was carried out using disposable gloves, sterilized standard mouth mirrors and probes and calipers. All occlusal relationships were evaluated at a centric occlusion position which was achieved by asking the child to swallow and then to bite in his or her most posterior teeth. Class of malocclusion in Angle’s system of classification, presence of variables like crossbite, open bite, deep bite, protrusion of teeth, tooth rotation, midline deviations and midline diastema were recorded. Children with class I molar relationship, normal overbite and overjet, proper alignment and no gross irregularities of tooth were categorized in normal occlusion group.

Personal data and previous history of orthodontic treatment were obtained directly from the children.

### Statistical Analysis

The prevalence of malocclusion was represented in proportions. Differences in proportion among the group were analyzed using Chi-square test and data were analyzed using statistical software Statistical Packages for the Social Sciences (SPSS); p values less than 0.05 were considered as statistically significant.

## RESULTS

Among the 2,366 children examined for the prevalence of malocclusion, 54.1% were boys and 45.9% were girls. The age and gender distribution of the children examined are shown in Table 1.

**Graph 1:**
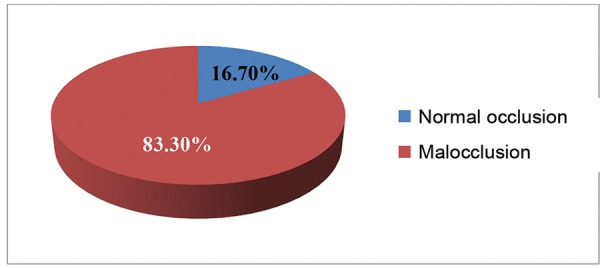
Prevalence of malocclusion

Among the children examined, 83.3% presented with malocclusions (Graph 1). To categorize the malocclusion in the examined children, 69.8% had class I malocclusion, 9.3% had class II malocclusion and 4.1% had class III malocclusion. Within class II malocclusion, 8.85% was division 1 type and 0.5% was division 2 type. The distribution of subjects based on gender and Angle’s class of malocclusion was not statistically significant (Table 2).

Of the 2,366 children who were examined for oveq’et, 64.1% had normal overjet (<3 mm) and 23.2% had an increased overjet (>3 mm). A small percentage (4.2%) had reverse overjet (Table 3).

Among the total children examined, 64.1% had normal overbite (<3 mm), 35.6% had an increased overbite (>3 mm) and a small percentage (0.29%) had open bite (Table 4). The gender distribution was statistically significant in this group. Among the total of seven children (0.29%) with open bite (Table 5), six were purely skeletal with a male:female ratio of 1:5 and only one male child presented with dental open bite.

To present the crossbite, among the total children examined, 7.2% had teeth in crossbite, of which 1.2% presented with complete crossbite, 4.6% had crossbite of complete anterior teeth, 66.3% were with single or multiple anterior teeth in crossbite, 18.6% had unilateral posterior teeth in crossbite and 9.3% had both anterior and unilateral posterior teeth in crossbite (Table 6). Among the single-tooth crossbite, the most frequently noted tooth was maxillary right lateral incisor (61.4%) followed by maxillary left lateral incisor (38.5%). Gender distribution was also significant as only male children had complete crossbite.

**Table 1:** **Table 1:** Descriptive statistics of the children examined

*Age (in years)*		*Male n (%)*		*Female n (%)*		*Total n (%)*	
10		358 (52.6)		*322 (47.4)*		680 (28.7)	
11		334 (53.7)		288 (46.3)		622 (26.3)	
12		589 (55.4)		*475* (44.6)		1064 (45)	
Total		1281 (54.1)		1085 (45.9)		2366 (100)	

In the examination of the midline with respect to the maxillary arch, 36.6% had no deviation, 30.23% had deviation to right and 33.17% had deviation to left (Table 7).

**Table 2:** **Table 2:** The classification of the subjects based on Angle’s class of malocclusion

*Occlusion*				*Male n (%)*		*Female n (%)*		*Total n (%)*			
Normal occlusion				187 (47.1)		210 (52.9)		397 (16.7)			
Class I malocclusion				911 (55.1)		741 (44.9)		1652 (69.8)			
Class II malocclusion		Div. 1		127 (60.8)		82 (39.2)		209 (8.85)		221 (9.3)	
		Div. 2		3 (25)		9 (75)		12 (0.5)		
Class III malocclusion		Class III		53 (55.2)		43 (44.8)		96 (4.1)			
Total				1281 (54.1)		1085 (45.9)		2366 (100)			

Chi-square = 2.249; p = 0.325

**Table 3:** **Table 3:** Distribution of overjet among the subjects

*Overjet*		*Male n (%)*		*Female n (%)*		*Total n (%)*	
Normal (<3 mm)		929 (51.4)		878 (48.65)		1807 (76.4)	
Increased (>3 mm)		346 (63.0)		203 (37.0)		549 (23.2)	
Reverse overjet		6 (60)		4 (40)		10 (0.4)	

Chi-square = 22.713; p = 0.000

**Table 4:** **Table 4:** Distribution of overbite among the subjects

*Overbite*		*Male n (%)*		*Female n (%)*		*Total n (%)*	
Normal (<3 mm)		775 (51.0)		742 (49)		1517 (64.1)	
Increased (>3 mm)		504 (59.9)		338 (40.1)		842 (35.6)	
Open bite		2 (28.5)		5(71.5)		7 (0.29)	

Chi-square = 17.199; p = 0.000

**Table 5:** **Table 5:** Distribution of open bite among the subjects

*Type of openbite*		*Male*		*Female*		*Total*	
Skeletal		1 (16.7%)		5 (83.3%)		6 (85.7)	
Dental		1 (100%)		–		1 (14.3%)	
Total		2 (28.5%)		5 (71.5%)		7 (100%)	

**Table 6:** **Table 6:** Distribution of crossbite among the subjects

*Type of crossbite*		*Male*		*Female*		*Total*	
Complete (anterior + bilateral posterior)		2 (100%)		–		2 (1.2%)	
Anterior Complete		4 (50%)		4 (50%)		8 (4.6%)	
Single tooth		62 (54.3%)		52 (45.6%)		114 (66.3%)	
Unilateral posterior		17 (53.1%)		15 (46.8%)		32 (18.6%)	
Anterior + unilateral posterior		10 (62.5%)		6 (37.5%)		16 (9.3%)	
Total		95 (55.23%)		77 (44.77%)		172 (100%)	

**Table 7:** **Table 7:** Distribution of midline deviation

*Midline*		*Male*		*Female*		*Total*	
No deviation		457 (52.7%)		409 (47.2%)		866 (36.6%)	
Deviated to right		382 (53.4%)		333 (46.6%)		715 (30.2%)	
Deviated to left		442 (56.3%)		343 (43.7%)		785 (33.2%)	

Chi-square = 2.283; p = 0.319

**Table 8:** **Table 8:** Distribution of midline diastema and rotation

*Variable*		*Male*		*Female*		*Total*	
Midline diastema		9 (50%)		9 (50%)		18 (0.76%)	
Tooth rotation		34 (44.2%)		43 (55.8%)		77 (3.25%)	

Among the children examined for midline diastema, 0.76% had midline diastema (Table 8) and it was completely in the maxillary arch.

Rotation of tooth was found to be the most common individual tooth irregularities in our study group. In the total group of children examined, 3.25% had rotation of tooth (Table 8). Lateral incisors (64.9%) were found to be the more frequently rotated type of teeth. The most rotated teeth were maxillary right lateral incisors (19.4%).

**Graph 2:**
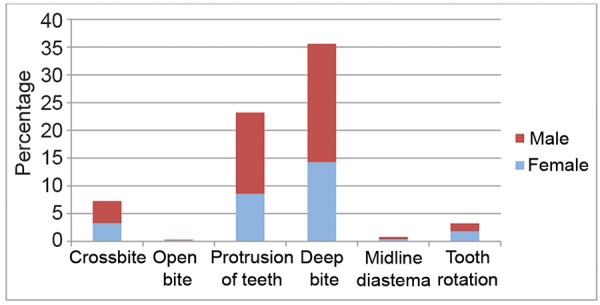
Prevalence and gender distribution of different variables of malocclusion

Overall distribution of the variables of malocclusion among subjects examined (Graph 2) revealed that deep bite (35.6%) was the most prevalent, followed by increased overjet (23.2%) and crossbite (7.2%). The least noted characteristics were open bite (0.29%) and midline diastema (0.76%).

## DISCUSSION

Malocclusion is one of the most common dental problems in mankind. Maloccluded teeth can cause psychosocial problems related to impaired dentofacial aesthetics, disturbances of oral function, such as mastication, swallowing and speech, and greater susceptibility to trauma and periodontal disease.

Numerous studies have been published regarding the prevalence of malocclusion in various populations. The results have shown wide variations. Differences in the age ranges of the populations studied, the number of subjects examined and differences in the registration methods are probably the most important factors explaining these variations.^5^

In the last decade, a number of studies have attempted to examine the malocclusion problem on a population basis using cross-sectional examinations of groups claimed to be representative of the Indian nation. But very few studies were known to be reported based on population of Kerala state of India. The present study was conducted among 2,366 schoolchildren aged 10-12 years to determine the prevalence of malocclusion in Kozhikode district of Kerala, India.

The present study evaluated the occlusion status of the subjects using Angle’s classification of malocclusion. Of the children examined in the present study, 16.7% of subjects reported with normal occlusion and 83.3% with malocclusions. The prevalence of malocclusion in our study is almost similar to the study done by Kaur et al^6^ in Karnataka, India (87.79%) and Ajayi^7^ in Nigeria (84.1%). When compared with our study, a higher prevalence of malocclusion was reported by Abu Alhaija et al^8^ in Jordan (92%) and Rwakatema^9^ in Tanzania (97.6%). But the studies conducted by Hemapriya et al^10^ in Kancheepuram (75%) and Trehan et al^11^ in Jaipur (66.7%) reported a lower prevalence than the present study.

The prevalence of class I malocclusion seen in the present study (69.8%) is almost similar to the findings by Trehan et al^11^ in Jaipur (57.9%) and Das and Reddy^12^ in Bengaluru (61.6%), India. This is found to be higher according to studies of Phaphe et al^13^ in Bagalkot (17.8%) and Vibhute et al^14^ in Mumbai (49.1%), India and lower when compared to the findings of Mtaya et al^5^ in Tanzania (93.6%) and Ajayi^7^ in Nigeria (80.7%). Class II malocclusion seen in the present study (9.03%) is similar with the findings of Sridharan et al^15^ in Tumkur (10%) and Muppa et al^16^ in Andhra Pradesh (9.95), India. This is found to be higher than the results of Mtaya et al^5^ in Tanzania (4.4%) and Shrestha et al^17^ in Kathmandu (2.5%) and was lower when compared with the studies of Abu Alhaija et al^8^ in Jordan (18.8%) and Phaphe et al^13^ in Bagalkot, India (30.1%). Class III malocclusion (4.1%) found in the present study was almost similar to that reported by Vibhute et al^14^ in Mumbai (5.7%), India and Thilander et al^18^ in Columbia (3.7%). This is found to be higher according to Abu Alhaija et al^8^ in Jordan (1.4%) and Das et al^12^ in Bengaluru, India (0.6%) and lower when compared with the findings of Farahani et al^1^ in Iran (7.8%) and Cedikoglu et al^19^ in Turkey (16.7%). There was no significant difference in gender distribution for the prevalence of different classes of malocclusion in our study.

In the present study, overjet less than 3 mm was categorized as normal and it was found that 23.2% of the subjects were with increased overjet, which is similar to the findings of Abu Alhaija et al^8^ in Jordan (24.7%) and Gelgor et al^20^ in Central Anatolia (25.1%). The studies by Farahani et al^1^ in Iran (31.7%) and Hemapriya et al^10^ in Kancheepuram, India (61.4%) reported a higher prevalence of increased overjet, while lower prevalence was reported in the studies by Siddegowda and Rani^21^ in Karnataka (6.3%), India and Poeung et al^22^ in Cambodia (8.1%).

In the present study, overbite less than 3 mm was categorized as normal and it was found that 38.0% of the subjects were with increased overbite, which is similar to the findings of Cedikoglu et al^19^ in Turkey (36.6%) and Nainani and Sugandh^23^ in Nagpur, India (38.0%). But the studies by Tausche^3^ in Dredsen (46.2%) and Siddegowda and Rani^21^ in Karnataka, India (51.75%) reported a higher prevalence of increased overbite, and studies by Rwakatema^9^ in Tanzania (20%) and Phaphe et al^13^ in Bagalkot, India (9.2%) reported a lower prevalence than that in the present study.

In our study, only 0.29% of the subjects reported with anterior open bite. The studies by Farahani et al^1^ in Iran (1.6%), Mtaya et al^5^ in Tanzania (1.8%) and Ciuffolo et al^24^ in Italy (1.7%) also reported a very low prevalence of open bite. When compared with our study, the findings of the studies conducted by Nainani and Sugandh^23^ in Nagpur (2.98%), India, Ajayi^7^ in Nigeria (4.1%) and Poeung et al^22^ in Cambodia (16.4%) were much higher. In the present study, it was found that 7.1% children had crossbite which corroborates with the studies by Nainani and Sugandh^23^ in Nagpur, India (5.5%) and Abu Alhaija et al^8^ in Jordan (6.7%). The studies by Siddegowda and Rani^21^ in Karnataka (18%), India and Poeung et al^22^ in Cambodia (14.7%) reported with a higher prevalence of crossbite. In our study, 4.2% reported with anterior crossbite, which is similar to the findings of Muppa et al^16^ in Andhra Pradesh (4.98), India and Bittencourt et al^25^ in Brazil (5%). But the studies by Phaphe et al^13^ in Bagalkot (7.2%), India and Cedikoglu et al^19^ in Turkey (14.1%) showed a higher value of crossbite.

A midline diastema is considered to be present when there is a space of at least 2 mm between the maxillary central incisors. Among the total subjects evaluated, only 0.76% were with maxillary midline diastema. This finding is very lower when compared with the studies by Phaphe et al^13^ in Bagalkot (18%), Hemapriya et al^10^ in Kancheepuram (35.2%), India and Ajayi^7^ in Nigeria (19.5%). The children reported with rotated tooth in the present study was 3.25%, which was lower when compared with the findings of Vibhute et al^14^ in Mumbai (13.1%) and Nainani and Sugandh^23^ in Nagpur (15.3%).

It is essential to identify and localize the wide range of deviations from occlusal development that may arise and that must be intercepted before the end of the active growth stage. Problems of a functional nature that arise from these morphological changes may become more complex skeletal problems in the future with serious psychosocial consequences for the developing individual.^4^

The findings of the present study will be very useful for the early interceptive measures as well as early correction of the malocclusion, thus reducing its severity in the permanent dentition.

## CONCLUSION

The following conclusions are drawn from the present study:

- Prevalence of malocclusion was found to be 83.3%. Class I malocclusion was the most prevalent type (69.8%), followed by class II malocclusion (9.3%), with 8.85% of division 1 type and 0.5% of division 2 type, and class III malocclusion (4.1%).
- Prevalence of normal occlusion was seen more in females, whereas prevalence of malocclusion was more in males. This difference was not statistically significant.
- The prevalence of crossbite and openbite was 7.2 and 0.29% respectively. Maxillary right lateral incisor was the most common tooth in crossbite.
- The prevalence of excessive overjet (protrusion of maxillary teeth) and excessive overbite (deep bite) was 23.2 and 35.6%, respectively.
- The prevalence of midline diastema was 0.76%.
- The prevalence of rotation was 3.25% and the most commonly rotated tooth was maxillary right lateral incisor followed by mandibular right lateral incisor.
- There was no statistically significant difference in midline deviation.

To conclude, the results of the present study confirmed that there is increased prevalence of malocclusion among children in the 10-12 years age group. The finding of this study will provide baseline data for implementing early interceptive treatment for the elimination of factors inhibiting dental arch development as well as skeletal jaw growth.
